# Bilateral blockade of MEK- and PI3K-mediated pathways downstream of mutant KRAS as a treatment approach for peritoneal mucinous malignancies

**DOI:** 10.1371/journal.pone.0179510

**Published:** 2017-06-22

**Authors:** Murali R. Kuracha, Peter Thomas, Brian W. Loggie, Venkatesh Govindarajan

**Affiliations:** 1 Department of Surgery, Creighton University, Omaha, Nebraska, United States of America; 2 Department of Biomedical Sciences, Creighton University, Omaha, Nebraska, United States of America; Duke University School of Medicine, UNITED STATES

## Abstract

Mucinous colorectal adenocarcinomas (MCAs) are clinically and morphologically distinct from nonmucinous colorectal cancers (CRCs), show a distinct spectrum of genetic alterations (higher KRAS mutations, lower p53, high MUC2), exhibit more aggressive behavior (more prone to peritoneal dissemination and lymph node involvement) and are associated with poorer response to chemotherapy with limited treatment options. Here, we report the effectiveness of combinatorial targeting of two KRAS-mediated parallel pathways in reducing MUC2 production and mucinous tumor growth in vitro and in vivo. By knockdown of mutant KRAS we show that, mutant KRAS (a) is necessary for MUC2 production in vitro and (b) synergistically engages PI3K/AKT and MEK/ERK pathways to maintain MUC2 expression in MCA cells. These results define a novel and a previously undescribed role for oncogenic KRAS in mucinous cancers. MCA cells were sensitive to MEK inhibition suggesting cellular dependence (‘addiction’) of KRAS-mutant MCA cells on hyperactivation of the MEK-driven pathway. Interestingly, MCA cells, though initially sensitive, were later resistant to PI3K single agent inhibition. Our studies suggest that this resistance involves dynamic rewiring of signaling circuits mediated through relief of RTK inhibition and MEK-ERK rebound activation. This resistance however, could be overcome by co-targeting of PI3K and MEK. Our studies thus provide a rational basis for MEK- and PI3K-targeted combination therapy for not only KRAS mutant MCA but also for other related mucinous neoplasms that overproduce MUC2 and have a high rate of KRAS mutations such as pseudomyxoma peritonei.

## Introduction

Sporadic colorectal cancers comprise a heterogeneous group of tumors and a significant proportion (10–15%) are of the mucinous subtype (defined as >50% of the tumor) [1]. MCAs are most frequently found in the proximal colon [2]. The etiology of MCA is poorly understood and a strong association has been shown with premalignant serrated neoplasms which are distinct from colonic adenomas [3]. MCAs show a different spectrum of molecular and genetic alterations than nonmucinous CRCs. In addition to excessive mucin production, MCAs are characterized by higher microsatellite instability (MSI-H), higher CpG Island Methylator Phenotype (CIMP-H) and higher LINE-1 methylation [4]. Recent studies report higher mutation rates for KRAS, BRAF and PIK3CA in MCA than for nonmucinous CRCs [5]. Clinically, MCAs present at a more advanced stage and are more prone to peritoneal dissemination and lymph node metastasis [2, 6–8]. MCAs generally have a poor prognosis; in advanced stage disease (stages III and IV) MCAs have been shown to have worse outcomes and poorer response to palliative chemotherapy compared to nonmucinous CRCs [6–8]. Pseudomyxoma peritonei (PMP) is a related mucinous neoplasm that arises in the appendix and is prone to peritoneal dissemination [9]. PMP presents as bulky, multifocal tumor implants within the peritoneal cavity and a characteristic feature of this malignancy is the overproduction of mucinous ascites in patients with advanced disease [10]. Treatment options for PMP patients are limited and cytoreductive surgery in association with hyperthermic intraperitoneal chemotherapy is the primary treatment modality. Similar to MCA, a high rate of KRAS mutations have been reported in PMP [11]. The higher prevalence of KRAS mutation in these mucinous cancers precludes EGFR inhibitor therapy as a treatment option [12] underscoring the need for identification of novel therapeutic targets.

MUC2, a secreted gel forming mucin, is abundantly expressed by goblet cells of the healthy colon and small intestine. MUC2 expression is seen in colon adenomas but in nonmucinous CRCs, MUC2 is reduced or lost during progression to adenocarcinoma [13]. In contrast, MUC2 expression is retained in MCAs [13] suggesting a potential role for MUC2 in the pathobiology of MCA. MUC2 overexpression or ectopic expression is a common property of all mucinous carcinomas including that of the ovary, breast, pancreas [14] and PMP [15] suggesting a common genetic alteration associated with the mucinous tumor phenotype. MUC2 overproduction is both pathognomonic and pathologic for peritoneal mucinous neoplasms such as PMP [15]. Though a high rate of KRAS mutations are seen in MCA and PMP, the question whether mutant KRAS oncoprotein is necessary to maintain expression of MUC2 in these mucinous malignancies has not been explored.

KRAS belongs to the RAS family of proteins and is among the most frequently activated drivers of human cancers [16]. Mutant RAS oncoprotein has been shown to activate a variety of signaling pathways downstream including the sequential activation of RAF, MEK and ERK to influence neoplastic growth [17]. Inhibitors targeting the effectors in the RAF/MEK/ERK signaling cascade have been approved for use (e.g. mutant BRAF inhibitor, Vemurafenib) [18] and others are in various stages of clinical development (e.g. MEK inhibitors Trametinib, Pimaretib etc.) [19].

Phosphatidylinositol 3-kinases (PI3K) are lipid kinases that are divided into three classes, class I, II and III. Of these class I PI3Ks are associated with cancer [20]. Class I PI3K, a heterodimeric protein, comprises a catalytic subunit ((p110), encoded by the gene PIK3CA) and a regulatory subunit (p85). PIK3CA mutations, commonly in the kinase domain (e.g. H1047R) or mutations in the helical domain that relieve inhibitory interaction with the regulatory subunit (e.g. E542K, E545K), lead to constitutive activation of PI3K and its downstream effectors such as AKT, mTORC1, S6 Kinase [21]. Through these signaling effectors, the PI3K signaling pathway regulates growth and survival [20]. More than 20 inhibitors targeting various nodes of the PI3K signaling pathway are in various phases of clinical development [22].

Currently, there are no diagnostic or targeted treatment interventions specific to mucinous malignancies such as MCA or PMP. Our studies reported here reveal MEK and PI3K as therapeutic targets for treatment of MCA and PMP. Our results suggest that, a) mutant KRAS, by synergistic engagement of MEK and PI3K pathways, maintains MUC2 expression and b) dual inhibition of MEK and PI3K is more effective in reducing tumor cell viability in vitro and in vivo than single agent treatment. These results thus provide a rational basis for combinatorial inhibition of MEK and PI3K in KRAS-mutant mucinous cancers derived from the colon or the appendix.

## Materials and methods

### Cell lines

LS174T [23] (American Type Culture Collection), RW7213 and RW2982 [24] cell lines were cultured in RPMI (Roswell Park Memorial Institute) 1640 (Hyclone Laboratories, Logan, UT) supplemented with 4.5 g/L glucose (Invitrogen), 10% FBS (Invitrogen), 2 mM L-glutamine, 20 mM HEPES, 1X MEAA, 100 IU/ml penicillin, and streptomycin in an incubator at 37°C with 5% CO_2_. These cell lines were tested for interspecies contamination and authenticated by short tandem repeat (STR) analysis using 16 STR markers (Idexx BioResearch, Columbia, MO).

### ShRNA knockdown studies

Short hairpin RNA directed against sequences specific to wildtype KRAS, mutant KRAS, HRAS or NRAS were designed (S1 Table), amplified and cloned into the pINDUCER10 lentiviral vector [25]. The pINDUCER lentiviral vectors containing shRNAs were co-transfected with lentiviral packaging plasmid vectors into 293T cells using Fugene HD (Promega, Madison, WI) transfecting reagent as previously described [25]. Three days after transfection, the viral particles were collected and concentrated through Amicon Ultra-15 (MWCO 100,000) (EMD Millipore, Billerica, Massachusetts) and used to infect LS174T or RW7213 cells in the presence of polybrene (9 μg/mL) for 48 h. Stable cell lines were established by Puromycin (2.5 μg/mL) (MP Biomedicals, Solon, OH) selection. Following selection and induction of tRFP expression by addition of doxycycline (1 μg/mL) for 24 hours, the top 10% of RFP^hi^ cells were sorted by FACS, clonally expanded and stable cell lines established. Non-silencing shRNA (SHC016, Sigma-Aldrich, St Louis, MO) was cloned into the pINDUCER vector, sorted and stable lines were generated as described above.

### Cell viability assays

Cells were plated in six replicate wells and treated with Cobimetinib (MEKi) (1.6 nM to 100 μM) or Pictilisib (PI3Ki) (16 nM to 1 mM) and cell viability was measured 96 hours later using the Wst1 Cell Viability Assay (Promega). The concentration of drug resulting in 50% of maximal inhibition (IC50) was calculated from a four-parameter sigmoidal dose response model (XLfit, IDBS). In vitro synergy studies were performed by addition of MEK and PI3K inhibitors in a fixed ratio ranging from 0.0039X to 4X IC50 of each drug, either alone or in combination. Synergy between drugs was assessed using Compusyn, a program that uses Chou and Talalay combination index (CI) for computing synergism, additivity or antagonism [26].

### Western blots

Cells were lysed in RIPA lysis buffer containing a protease phosphatase cocktail inhibitor (Cell signaling, Danvers, MA). After centrifugation at 2000g at 4°C, the supernatant was mixed with equal volume of sample buffer (4% SDS, 10% β- mercaptoethanol, 20% glycerol, 0.5M Tris-Hcl pH 6.8, 0.05% Bromophenol blue) and denatured. Proteins were resolved by 4–15% SDS-PAGE (KRAS, pERK, tERK, pAKT, tAKT, MEK, pS6, cleaved caspase 3) or by agarose (1.5%) gel electrophoresis (MUC2). Proteins were electrotransferred to PVDF membrane (Millipore). After transfer, the blots were blocked for one hour at room temperature and probed with one of the following antibodies overnight at 4°C; anti-KRAS (cat# H0003845 Abnova, Taipei, Taiwan) (detects both wildtype and mutant KRAS), anti-KRASG12D (cat# D8H7, Cell Signaling, Danvers, MA) (detects only mutant KRAS), anti- Mucin2 (cat# 550485, BD Biosciences, San Jose, CA), anti-β actin (cat# A5441, Sigma Aldrich), phosphorylated ERK (pERK) (cat# 4376, Cell signaling), total ERK (tERK) (cat # 4695, Cell signaling), phosphorylated AKT (pAKT) (cat# 4060, Cell signaling), total AKT (tAKT) (cat # 4691, Cell signaling), RS6K (cat # 2211, Cell signaling) and cleaved caspase 3 (cat# 9664, Cell signaling). The blots were then washed three times and incubated with the appropriate secondary antibody for one hour at room temperature. The antigen- antibody complex was detected by enhanced chemiluminescence (Super Signal West Pico, Pierce Thermo Scientific, Waltham, Massachusetts) according to manufacturer’s instructions and the blots were then exposed to x-ray film. Signals on the blots were quantified by densitometry (Quantity one 4.6.5, Bio-Rad, Hercules, CA). All the western blots were performed at least twice. Due to its high molecular weight and glycosylation MUC2 was resolved by 1.5% agarose gels and the respective β-actin loading controls were resolved by conventional SDS-PAGE gels.

### RTK arrays

Human Phospho-RTK arrays (ARY001B, R&D systems, Minneapolis, MN) were used to assess alterations in phosphorylation of RTKs in response to PI3K inhibition. Briefly, LS174T or RW7213 cells were treated with 3.0 μM or 5.6 μM Pictilisib respectively for 72 hours. Cells were then washed with cold PBS, lysed in NP40 lysis buffer, and 300 μg of cell lysates were incubated with blocked membranes overnight. Membranes were subsequently washed and incubated with an HRP-conjugated anti-phosphotyrosine antibody (supplied with the kit), for 2 hours at room temperature, rinsed in a wash buffer (supplied with the kit) and incubated with a chemiluminescent substrate according to manufacturer’s instructions. The membrane was then exposed to X-ray film and the results quantified by densitometry.

### Quantitative real time PCR (QPCR)

HRAS and NRAS knockdown in MCA cells were assessed by quantitative real time RTPCR (qRTPCR). Total RNA was extracted from doxycycline-treated or untreated LS174T or RW7213 cells using Totally RNA isolation kit (AM1910, Ambion, Austin, TX). RNA was treated with RNase-free DNase (Invitrogen, Carlsbad, CA) for 30 min at room temperature and first strand cDNA was synthesized from 1 μg of DNase-treated RNA by MMLV reverse transcriptase (New England Biolabs, Ipswich, MA). For qRTPCR, 1μl of cDNA was mixed with 10 μl of 2 X Fast SYBR green mix (Applied Biosystems, Foster, CA), oligonucleotides and water was added to the premade 96 well plates and real time PCR was run on a PRISM 7500 machine (Applied Biosystems). The oligonucleotides used for amplification include 5’CAGTCGCGCCTGTGAACGGTGG 3’ and 5’CCAGCTTATATTCCGTCATCG 3’ for HRAS and 5’ GCTTCCTCTGTGTATTTGCCA 3’ and 5’ GCACCATAGGTACATCATCCG 3’ for NRAS. GAPDH (control) was amplified using primers 5’AATGAAGGGGTCATTGATGG 3’ and 5’AAGGTGAAGGTCGGAGTCAA 3’. In order to rule out probable contamination of genomic DNA, negative controls were performed in parallel by directly using RNA as a template for PCR.

### Animal studies

Four to six-week-old homozygous female nude mice (Crl:NU (NCr)-Foxn1^nu^) (Charles River labs; Wilmington, MA) were used for in vivo studies. Animal studies were performed in accordance with the recommendations in the Guide for the Care and Use of Laboratory Animals of the NIH. All the mouse studies described here were approved by the Institutional Animal Care and Use Committee at Creighton University. Tumors were induced by subcutaneous injections of LS174T cells and once tumors reached a volume 200 mm^3^, mice were randomized into four groups (vehicle, MEKi, PI3Ki and MEKi+PI3Ki) with an n = 8 for each group. Cobimetinib (MEKi) (20 mg/kg body weight) and Pictilisib (PI3Ki) (150 mg/kg bodyweight) were given by oral gavage once in three days (Q3D). Tumor volume was measured using the formula (length x (width)^2^/2). Tumor growth inhibition was measured by calculating the area under the curve (AUC) (XLfit, IDBS). Upon initiation of tumor growth, animals were monitored daily for alterations in body weight, behavior (ability to reach for food and water, breathing, signs of lethargy or lack of physical activity, lack of grooming behavior), physiology (abdominal distension, mucous discharge, signs of neurological deficits, dehydration) and body condition score [27]. At the end of the study, animals were euthanized by carbon dioxide inhalation followed by cervical dislocation. Animal studies were stopped at day 12 as the control group animals had reached humane end points. After the mice were euthanized, tumors were harvested for protein isolation and western blots. Tumor growth rates between different groups were compared by two-way ANOVA using GraphPad Prism 5.04 (GraphPad Software Inc., La Jolla, CA). A p<0.05 (two tailed) was considered significant.

## Results

### Mutant KRAS is necessary for MUC2 expression in MCA

**Fig 1:**
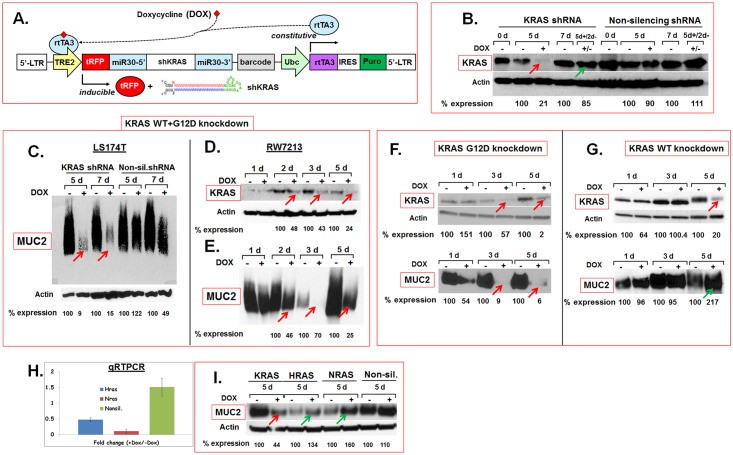
Inducible and reversible knockdown of mutant and wildtype KRAS in vitro. **A.** The pINDUCER lentiviral vector was used for targeted knockdown KRAS in MCA cells in vitro. Addition (+) or withdrawal (-) of DOX induced or extinguished respectively, the expression of KRAS shRNA (Fig adapted from Meerbrey et al. 2011). **B.** Western blots showing reduction of KRAS protein expression after induction of shRNA targeting wildtype and mutant KRAS in LS174T cells (red arrow) and restoration of KRAS protein expression within 2 days after DOX withdrawal (green arrow). Note that the KRAS western blot shown in panel B was probed with an anti-KRAS antibody that binds to both wildtype and mutant KRAS. **C-E.** KRAS knockdown reduces MUC2 protein expression in MCA in vitro. Knockdown of wildtype and mutant KRAS in LS174T (B) and RW7213 (D) cells reduces MUC2 protein expression (C, E). Note that the KRAS western blot shown in panel D was probed with an anti-KRAS antibody that binds to both wildtype and mutant KRAS. **F, G.** Knockdown of mutant but not wild type KRAS reduces MUC2. Western blots of mutant KRAS (F), wildtype KRAS (G) and MUC2 protein (F, G, bottom panels) expression in LS174T cells. Mutant KRAS knockdown in LS174T cells (F, top panel, arrows) substantially reduces MUC2 protein levels (F, bottom panel, arrows). In contrast, knockdown of wildtype KRAS (G, top panel, arrow) did not reduce MUC2 protein expression (G, bottom panel, arrow). Note that the KRAS western blot presented in panel F was probed with an anti-KRASG12D specific antibody that binds only to mutant KRAS protein while the western blot in panel G was probed with an antibody that binds both wildtype and mutant KRAS proteins. Knockdown of wildtype KRAS did not alter mutant KRAS protein levels (see S3 Fig). **H, I.** HRAS and NRAS are dispensable for MUC2 expression. Quantitative RTPCR results show reduction of HRAS and NRAS in LS174T cells (H). Error bars indicate standard error of the mean. Knockdown of HRAS or NRAS did not reduce MUC2 protein expression (I, green arrows) in contrast to KRAS knockdown (I, red arrow). Expression levels of proteins on western blots were quantified by densitometry and percent protein (MUC2 or KRAS) expression in DOX-treated (+) samples relative to untreated (-) controls (normalized to actin) are shown below the blots. Abbreviations: d, days; 5d+/2d- indicates that cells were cultured for 5 days in the presence of DOX and for 2 days after the removal of DOX.

Higher KRAS mutation rates reported in mucinous malignancies such as MCA and PMP correlate mutant KRAS oncoprotein to overproduction of MUC2, the predominant component of mucin, in these malignancies. In order to test the hypothesis that mutant KRAS is necessary for maintenance of MUC2 expression, mutant KRAS protein levels were reduced in two MCA cell lines, LS174T (KRASG12D) and RW7213 (KRASG12C), using short hairpin RNA (shRNA) targeting KRAS (Fig 1A). As these cells are heterozygous for mutant KRAS, we knocked down mutant KRAS and wildtype KRAS either singly (Fig 1F and 1G) or in combination (Fig 1B and 1D). Non-silencing shRNA were used as controls. Stable lines carrying shRNA targeting wildtype, mutant KRAS or both were established using the pINDUCER lentiviral system [25]. ShRNA expression in these lines was turned on or off by addition or withdrawal of DOX respectively (Fig 1A). Induction of KRAS shRNA by addition of DOX reduced KRAS protein levels (Fig 1B, red arrow) and withdrawal of DOX restored KRAS expression to almost normal levels within 48 hours (Fig 1B, green arrow) suggesting inducible and reversible knockdown of KRAS in these cells (note that the KRAS western blots shown in Fig 1B and 1D were probed with an anti-KRAS antibody that binds to both wildtype and mutant KRAS). Knockdown of both wildtype and mutant KRAS (Fig 1B and 1D) reduced MUC2 expression in LS174T (Fig 1C) and in RW7213 cells (Fig 1E). Similar reduction in MUC2 expression was seen with knockdown of mutant KRAS alone (Fig 1F, red arrows) (note that the KRAS western blot shown in Fig 1F was probed with an anti-KRASG12D-specific antibody that binds only to mutant KRAS protein). Interestingly, knockdown of wildtype KRAS alone (Fig 1G, red arrow) did not reduce MUC2 protein expression (Fig 1G, green arrow) (note that the KRAS western blot shown in Fig 1G was probed with an antibody that binds both wildtype and mutant KRAS proteins). In addition, knockdown of two related Ras proteins, HRAS and NRAS (Fig 1H), did not reduce, but modestly increased, MUC2 expression (Fig 1I, green arrows). Together, these results suggested that a) mutant KRAS is necessary for maintenance of MUC2 expression and b) wildtype KRAS, HRAS and NRAS were dispensable for MUC2 expression in MCA in vitro.

### Regulation of MUC2 expression by PI3K- and MEK- driven pathways downstream of mutant KRAS in MCA

**Fig 2:**
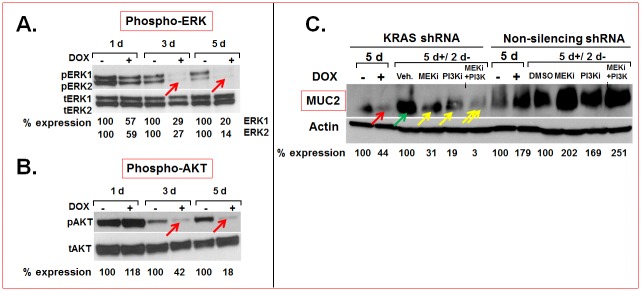
**A, B.** MEK and PI3K-driven pathways downstream of mutant KRAS maintain MUC2 expression in MCA. Mutant KRAS knockdown in LS174T cells reduced pERK1, pERK2 (A, red arrows) and pAKT (B, red arrows) protein levels. **C.** Mutant KRAS knockdown reduced MUC2 protein expression (red arrow) and removal of DOX from the media restored mutant KRAS expression (see Fig 1B) and MUC2 expression (C, green arrow). This recovery of MUC2 expression was blocked by treatment with either Cobimetinib (MEKi) or Pictilisib (PI3Ki) (C, single yellow arrows). Combined treatment with Cobimetinib and Pictilisib resulted in synergistic inhibition of MUC2 expression (C, double arrows). Non silencing RNAs were used as controls. Expression levels were quantified by densitometry and percentage protein expression in DOX-treated (+) samples relative to untreated (-) controls (normalized to actin) are shown below the blots. Abbreviations: d, days; 5d+/2d- indicates that the cells were cultured for 5 days in the presence of DOX and for 2 days after the removal of DOX.

As pharmacological inhibitors of mutant KRAS were not available, we attempted to define signaling effectors downstream of mutant KRAS that are necessary to maintain MUC2 expression and are more amenable to targeted therapy. Knockdown of mutant KRAS in MCA cells in vitro reduced phosphorylated ERK (Fig 2A, arrows) and AKT (Fig 2B, arrows) suggesting that PI3K-AKT and MEK-ERK driven pathways are activated downstream of mutant KRAS. In order to test the hypothesis that these signaling pathways regulate MUC2 expression downstream of mutant KRAS, we inactivated PI3K and MEK using small molecule inhibitors Pictilisib (GDC0941 or PI3Ki) and Cobimetinib (GDC0973 or MEKi) respectively. Pictilisib is a potent inhibitor of class I PI3K isoforms (p110α, β, ϐ, γ) and has been shown to be selective to PI3K when tested against 228 kinases [28]. Cobimetinib has been shown to inhibit MEK1 (IC50: 4.2 nM) with a 100-fold selectivity for MEK1 compared to 100 other serine-threonine kinases [29]. The PI3K and MEK-driven pathways were inactivated in stable lines that carried mutant KRAS shRNA that could be reversibly induced or extinguished by addition or withdrawal respectively of DOX (Fig 2C). This particular approach was taken in order to test whether a) these pathways are necessary for MUC2 expression and b) if these pathways are engaged downstream of mutant KRAS in regulation of MUC2 expression (direct treatment with MEK- or PI3K inhibitors would only test the former but not the latter). DOX-induced KRAS shRNA expression reduced KRAS protein levels (Fig 1B, red arrow) and in turn, MUC2 expression (Fig 2C, red arrow). Removal of DOX restored KRAS expression (Fig 1B, green arrow) and in turn, allowed the recovery of MUC2 protein expression (Fig 2C, green arrow). This recovery was blocked by addition of Pictilisib or Cobimetinib (Fig 2C, yellow single arrows). Interestingly, combined treatment of Pictilisib and Cobimetinib resulted in more substantial reduction of MUC2 protein expression than addition of either inhibitor alone (Fig 2C, yellow double arrows). These results suggested that both PI3K and MEK-driven pathways are synergistically engaged downstream of mutant KRAS to maintain MUC2 expression in MCA in vitro.

### Mutant KRAS and KRAS-driven pathways and MCA cell viability

**Fig 3:**
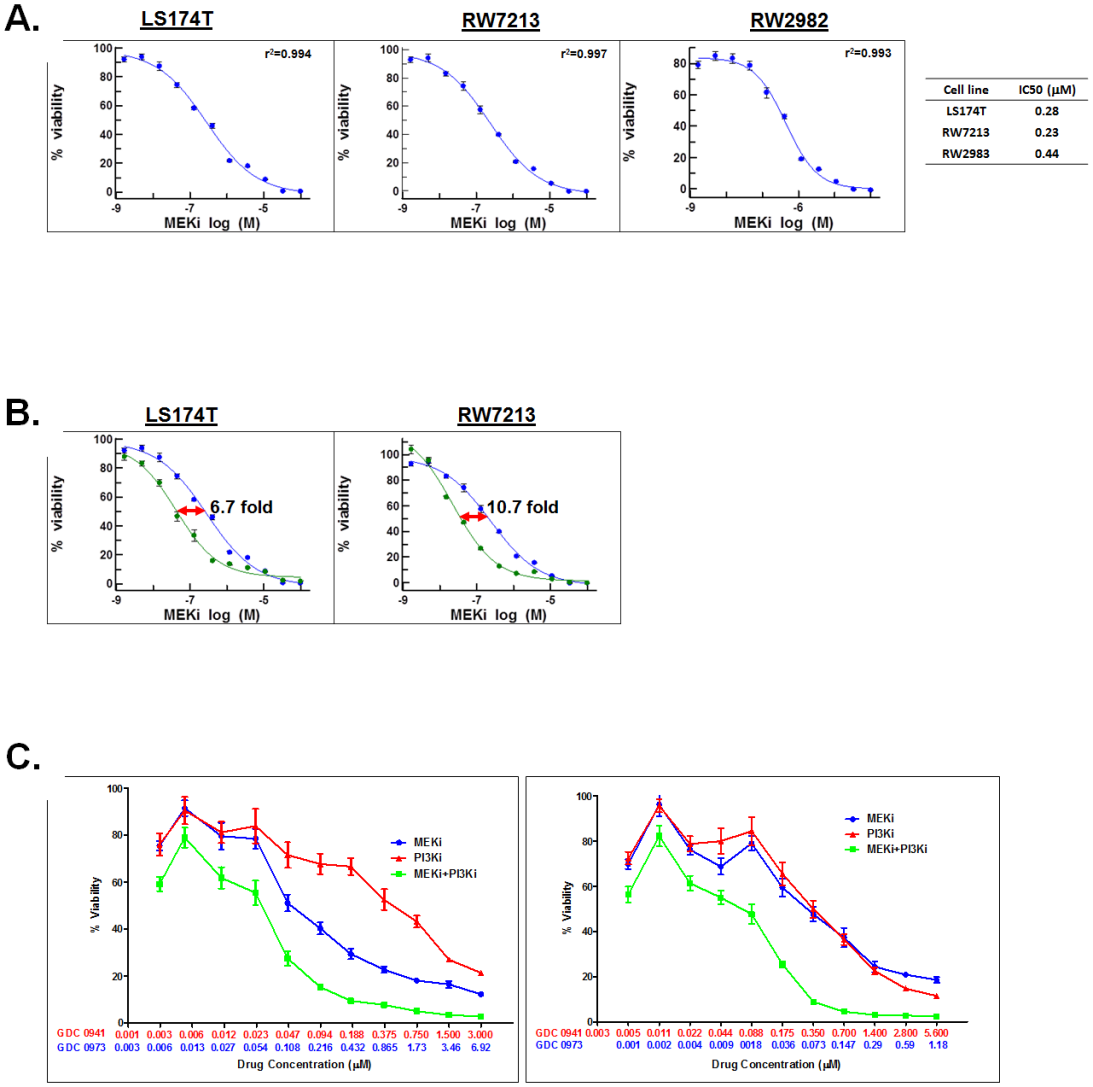
MCA cells are sensitive to MEK inhibition in vitro. **A.** LS174T, RW7213 and RW2982 cells were treated with increasing concentrations of the MEK inhibitor, Cobimetinib, for 96 hours. **B.** Mutant KRAS knockdown sensitizes MCA cells to MEK inhibition. LS174T and RW7213 cells, either untreated (blue) or induced with DOX (green) to knockdown mutant KRAS were treated with increasing concentrations of Cobimetinib (MEKi). The half-maximal concentration (IC50) of Cobimetinib was reduced from 0.28 μM to 0.042 μM (6.7 fold) in LS174T cells and from 0.24 μM to 0.022 μM (10.7 fold) in RW7213 cells. **C.** MEKi and PI3Ki combination treatment synergistically reduces viability of MCA in vitro. LS174T and RW7213 cells were treated with MEKi (blue) and PI3Ki (red) as single agents or in combination (green) in a fixed ratio for 72 hours. Each data point on all graphs is the average of an n = 6. Error bars indicate standard error of the mean.

Higher mutation rates reported in the effectors of RAS-RAF-ERK and PI3K-AKT pathways in MCA patients [5] suggested a cellular dependence of MCA on hyperactivation of these signaling pathways. In order to test this hypothesis, MCA cells were treated with increasing concentrations of Cobimetinib or Pictilisib. MCA cell lines, LS174T (KRASG12D), RW7213 (KRASG12C) and RW2982 (KRASQ61R) were sensitive to Cobimetinib (Fig 3A) with IC50 values of 0.24 μM, 0.23 μM and 0.44 μM respectively. Interestingly, in LS174T and RW7213 cells, initial knockdown of mutant KRAS followed by treatment with Cobimetinib increased the sensitivity of these cells to the drug by 6.7 and 10.7 fold respectively (Fig 3B) suggesting that reduction in mutant KRAS-mediated signaling allows better response to MEK inhibition. Sensitivity to Cobimetinib upon KRAS knockdown was not tested in RW2982 cells. Interestingly, MCA cell lines, LS174T and RW7213, though initially sensitive to PI3K single agent inhibition at 48 hours (S1 Fig), were resistant by 72 hours (data not shown) (i.e. the data (10 data points for each assay, n = 6 for each point) could not be fit to a four parameter logistic/sigmoidal dose response curve with an r^2^>0.9 and a negative Hill slope and an IC50 value could not be determined). But combined treatment of Cobimetinib and Pictilisib synergistically reduced viability of LS174T and RW7213 lines (Fig 3C). A combination index (CI) [26] of 0.421 (LS174T) and 0.417 (RW7213) suggested synergy between the two drugs in reducing MCA viability in vitro. These results suggested that the resistance to PI3K single agent inhibition seen in MCA cells could be overcome by MEKi and PI3Ki combination therapy. Increased cleavage of caspase 3, a hall mark of apoptosis, was seen in response to inhibitor treatment in MCA cells (S4 Fig) suggesting that the cytotoxic response to drug treatment was due to induction of apoptosis.

### Resistance to PI3Ki is correlated to pERK activation

**Fig 4:**
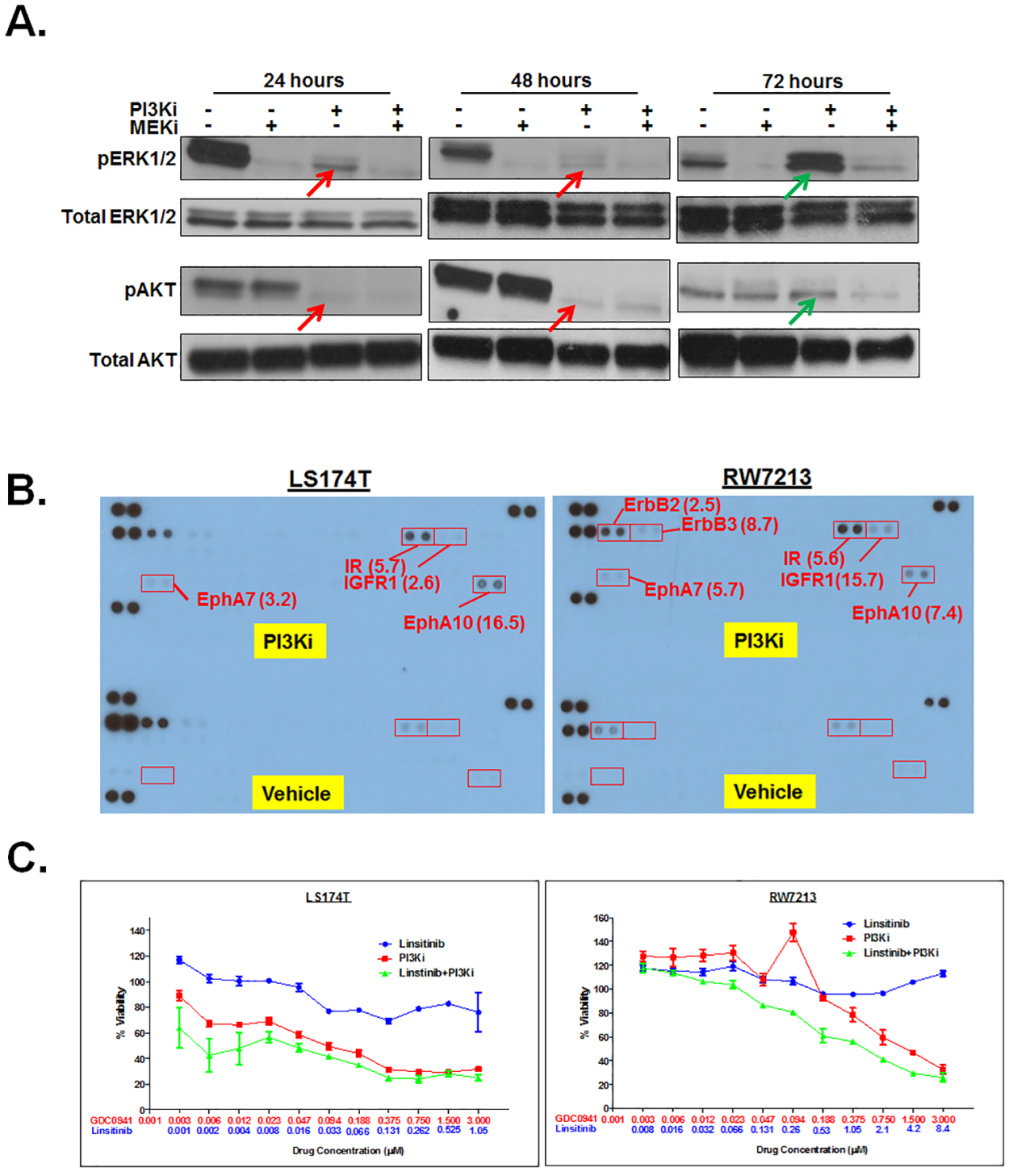
**A.** Resistance to PI3Ki is correlated to increased pERK. Western blots of proteins isolated from LS174T cells treated with either MEKi, PI3Ki or both were probed with anti-phosphoERK1/2 (pERK1/2), anti-total ERK1/2, anti-phosphoAKT (pAKT) or anti-total AKT. Elevated levels of pERK1/2 (A, green arrow) and pAKT (A, green arrow) were seen 72 hours after exposure to PI3Ki coincident with the development of resistance to this inhibitor. **B.** Increased receptor tyrosine kinase (RTK) phosphorylation upon prolonged exposure to PI3K inhibitors. Protein lysates from PI3Ki-treated LS174T or RW7213 cells (72 hours) were incubated with human phospho-RTK array containing 49 RTKs, washed and probed with anti-phospho-tyrosine antibodies. Antigen-antibody complexes were detected by chemiluminescence and the results were quantified by densitometry. Numbers within brackets indicate fold increase in RTKs in PI3Ki-treated MCA cells relative to vehicle-treated controls. **C.** Synergistic reduction in viability of MCA cells treated with PI3K inhibitor and Linsitinib (inhibitor of IR and IGFR1). LS174T and RW7213 cells were treated with Linsitinib (blue) and PI3Ki (red) as single agents or in combination (green) in a fixed ratio for 72 hours. Each data point is the average of an n = 6. Error bars indicate standard error of the mean.

In order to define the mechanistic basis of resistance of MCA cells to PI3K single agent inhibition but sensitivity to PI3Ki and MEKi combination therapy, we examined alterations in activation of downstream signaling effectors of PI3K and MEK-driven pathways. Phosphorylated AKT (pAKT), though initially reduced in MCA cells 24 and 48 hours (Fig 4A, red arrows) after treatment with Pictilisib (PI3Ki), was increased at 72 hours (Fig 4A, green arrow). Interestingly, a concomitant elevation in phosphorylated ERK (pERK) levels was also seen (Fig 4A, green arrows). However, the combined treatment with Cobimetinib and Pictilisib blocked this increase (Fig 4A). Similar changes were also seen in RW7213 cells (data not shown). Thus, resistance of MCA cells to PI3Ki single agent treatment but sensitivity to MEKi and PI3Ki combination therapy could be correlated to rebound activation of ERK.

### Relief of negative feedback of RTKs drives resistance to PI3Ki

The compensatory activation of MEK-ERK signaling in response to PI3K inhibition led us to investigate activation of receptor tyrosine kinase (RTKs) (transmembrane proteins) and upstream initiators of these signaling pathways. An anti-phospho-RTK array was used to assess alterations in phosphorylation status of 49 RTKs in response to PI3K single agent inhibition. In LS174T and RW7213 cell lines, 72 hours after PI3K inhibitor treatment (coincident with development of resistance), increases in phosphorylated insulin receptor (IR) (5.7 and 5.6 fold), insulin growth factor receptor 1 (IGFR1) (2.6 and 15.7 fold), Ephrin receptor A10 (EphA10) (16.5 and 7.4 fold) and Ephrin receptor A7 (EphA7) (3.2 and 5.7 fold) were seen relative to vehicle-treated controls (Fig 4B). In RW7213, additional increases in ErbB2 (HER2) (2.5 fold) and ErbB3 (HER3) (8.7 fold) were also seen (Fig 4B). In order to test the biological relevance of upregulation of these RTKs in mediating resistance to PI3Ki, MCA cells were treated with the PI3K inhibitor and Linsitinib (IR and IGFR1 inhibitor) [30]. This combination treatment resulted in synergistic loss of viability in LS174T and RW7213 cell lines (Fig 4C, green curves). As RW7213 also showed rebound activation of ErbB2 and ErbB3, this line was treated with the PI3K inhibitor and Lapatinib (inhibitor of EGFR and ErbB2 [31]; ErbB3 is kinase-impaired and forms active heterodimers with its preferred partner, ErbB2)). However, synergism was not seen between PI3Ki and Lapatinib in this line (S2 Fig). As there are no inhibitors currently available to target EphA7 and EphA10, the role of these RTKs in mediating PI3Ki resistance were not tested. Taken together, these results show that the development of resistance to PI3K inhibition in MCA cells is due to increased activation of RTKs, particularly IR and IGFR1.

### MEK and PI3K inhibition reduces MCA tumor growth in vivo

**Fig 5:**
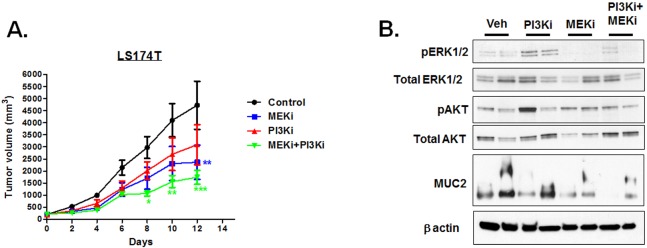
Combined treatment of Cobimetinib and Pictilisib is effective in reducing tumor growth in MCA xenograft mouse models. **A.** LS174T cell line-derived tumors in xenograft mouse models were treated with Cobimetinib (20 mg/kg) or Pictilisib (150 mg/kg) alone or in combination. Drugs were delivered Q3D by oral gavage. Tumor growth rates between different groups were compared by two-way ANOVA (two tailed). In the Fig, one, two and three asterisks indicate p<0.05, p<0.01 and p<0.001 respectively. **B.** Proteins from two tumors in each treatment group were resolved by SDS-PAGE, blotted and probed with antibodies to antigens indicated to the left of the blots. For detection of MUC2, proteins were resolved by 1.5% agarose gel. Combined treatment of MEKi and PI3Ki was effective in reducing MUC2 protein levels compared to single agent treatment.

In order to test the effectiveness of MEK and PI3K inhibitors in reducing MCA tumor growth in vivo, we generated subcutaneous tumors derived from LS174T cells in nude mice. Tumor-bearing mice were treated with Pictilisib or Cobimetinib. These drugs were given either alone or in combination and their effects on tumor growth and volume were compared to vehicle-treated controls. The drugs were administered by oral gavage Q3D (once in three days). This intermittent dosing regimen was chosen based on a previously published report that describes the effectiveness of these inhibitors on KRAS-mutant and BRAF-mutant non-small lung cancer and colorectal cancer lines [29]. MEK and PI3K single agent treatment resulted in a tumor growth inhibition (area under the curve) of 44.6% and 24.4% respectively while the combination treatment resulted in ~60% tumor growth inhibition (Fig 5A). Significant differences in tumor volumes were seen in Cobimetinib single agent treatment and Cobimetinib/Pictilisib combined treatment but not in the Pictilisib single agent treatment group relative to vehicle-treated controls (Fig 5A). These results were consistent with our in vitro results that show the MCA cells are resistant to PI3K, but not MEK, inhibition. Reduction in pERK levels could be seen in the tumors of MEKi and MEKi+Pi3Ki treatment groups (Fig 5B). But pAKT levels were not appreciably reduced in the PI3Ki treatment groups (Fig 5B) consistent with the rebound activation of pAKT in response to prolonged treatment with PI3Ki inhibitor (Fig 4A). Combinatorial inhibition of MEKi and PI3Ki substantially reduced MUC2 protein levels in vivo compared to single agent treatment and vehicle-treated controls (Fig 5B). Considered together, these results were consistent with our in vitro results that indicate that MEK and PI3K combination therapy is more effective than single agent therapy in reducing mucin overproduction and MCA cell viability.

## Discussion

Mucinous adenocarcinomas of the colon and rectum are generally proximal, right sided and show a distinct spectrum of genetic alterations and pathobiology [2]. These cancers likely originate as serrated adenomas and are driven by mutant KRAS or mutant BRAF rather than the conventional APC-KRAS-p53 pathway-driven cancers that originate in the distal colon [4, 5]. These mutant KRAS or BRAF-driven cancers show a poorer response to chemotherapy and are negatively indicated for treatment with EGFR inhibitors [12]. PMP, a related malignancy that arises from appendiceal neoplasms is also characterized by the production of excessive mucinous ascites (leading to bowel obstruction in advanced disease, the primary cause of patient mortality) and a high rate of KRAS mutations [9, 11]. Though MUC2 has been shown to be the predominant mucin family member in these cancers, the upstream signaling initiators of MUC2 expression in these malignancies are poorly understood. Though the relevance of the MEK-ERK and PI3K-AKT signaling pathways to MUC2 gene expression has been previously shown in colonic goblet cells and in colon cancer cells [32], the relevance of KRAS oncoprotein to MUC2 gene expression has not been directly assessed. The mutant KRAS knockdown studies we describe in this report reveal a novel and a critical role for this oncoprotein in regulating MUC2 expression in MCA cells in vitro. Interestingly, wildtype KRAS and two other related RAS proteins, HRAS and NRAS, were dispensable for maintenance of MUC2 expression. These results point to a unique biological role for the KRAS oncoprotein that does not overlap with the functions of other related RAS isoforms. These results are broadly consistent with previously published studies. For instance, the biological activities of the three RAS genes differ in regulating growth and differentiation of endodermal progenitors; activated HRAS promotes growth arrest and differentiation but activated KRAS promotes proliferation [33]. A more recent study indicates that KRAS mediates its functions by binding to and engaging effectors such as calmodulin (CaM), that are not bound by HRAS or NRAS [34]. Whether mutant KRAS-mediated regulation of MUC2 gene expression in MCA is a consequence of unique engagement of effectors by mutant KRAS, but not by HRAS or NRAS, is unclear and will need to be explored. Our data however, indicate that mutant KRAS synergistically utilizes both MEK-ERK and PI3K-AKT pathways to maintain MUC2 expression in MCA in vitro. Our results also suggest that combinatorial inhibition of these two signaling pathways is likely to be more effective than single agent therapy in reducing MUC2 expression in MCA and PMP. These results are also consistent with a recently published study that showed the effectiveness of RDEA119, a MEK inhibitor, in reducing MUC2 expression and mucinous tumor growth in in a patient-derived xenograft mouse model of pseudomyxoma peritonei, a mucinous malignancy that arises from the appendix [35]. It should be noted that the biological role of MUC2 in driving neoplastic growth of mucinous peritoneal malignancies such as PMP or MCA (i.e. as a driver oncogene) is not clear although recent studies indicate that mucin could play a role in increased dissemination and redistribution [36].

Increased mutation rates have been reported in the effectors of the RAS-RAF-MEK-ERK and PI3K-AKT pathways in MCA patients [5]. Our data suggest that MCA cell lines show a cellular dependence or ‘addiction’ to sustained activation of the RAS-RAF-MEK-ERK signaling pathway. KRAS mutant MCA cells appear to have a higher basal activation of the MEK-ERK signaling pathway likely due to constitutive activation of the KRAS oncoprotein. The 6–10 fold increase in sensitivity of MCA cells to MEK inhibition upon mutant KRAS knockdown supports this hypothesis. Our results are also consistent with previous reports that underscore the primacy of the RAS-RAF-MEK-ERK pathway in CRCs. For instance, Vemurafenib (BRAF inhibitor) elicited only a 5% response rate in BRAF-mutant CRC patients in contrast to dramatic responses seen in BRAF-mutant melanoma patients [37]. In another study, though BRAF-mutant CRCs were more sensitive to Dabrafenib (BRAF inhibitor) and Trametinib (MEK inhibitor) combination therapy than to Dabrafenib (BRAF inhibitor) single agent therapy, the response seen was less impressive than what has been observed in melanoma [38]. Rapid reactivation of ERK through EGFR-mediated activation of RAS and CRAF has been shown to be a reason for the diminished response seen to BRAF inhibitor in BRAF-mutant CRCs [39].

**Fig 6:**
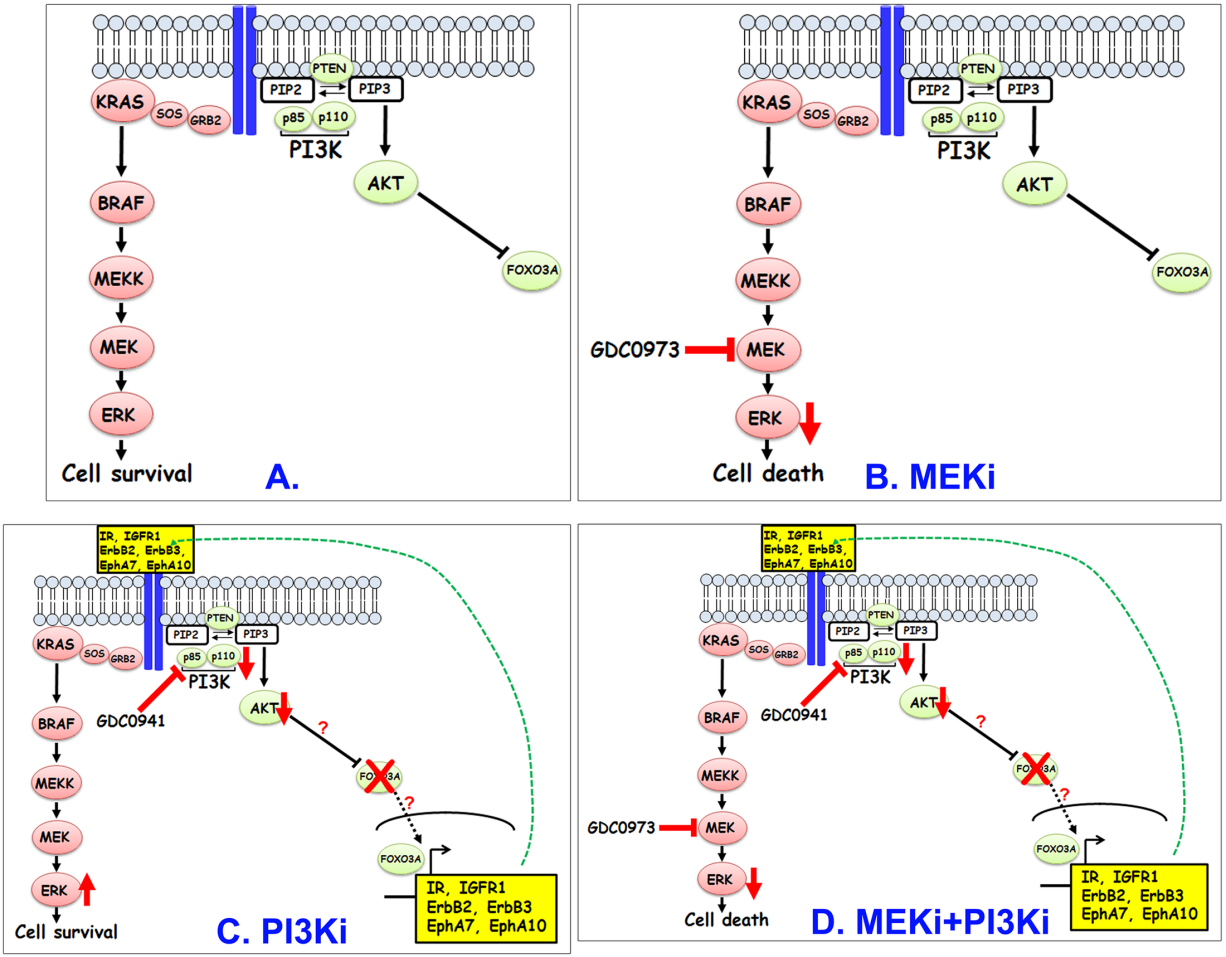
A model to explain the enhanced effectiveness of MEKi and PI3Ki in reducing viability of KRAS-mutant MCA cells. MEKi treatment (B) reduces pERK levels and leads to increased cell death. In contrast, PI3K inhibition (C) initially reduces AKT signaling but this reduction leads to enhanced expression of RTKs including IR, IGFR1, EphA10, EphA7, ErbB2 and ErbB3 presumably through relief of inhibition of FOXO proteins. Increased expression of these RTKs leads to their increased activation (likely through receptor homo- or heterodimerization) which in turn, stimulates rebound activation of MEK-ERK pathway which in turn, enhances survival and development of resistance. Combined inhibition of PI3K and MEK (D) prevents rebound activation of ERK in response to PI3K single agent treatment and helps overcomes resistance.

MCA cells, though initially sensitive, later became resistant to PI3K inhibition. Our results support a model (Fig 6) for resistance of MCA cells to PI3K inhibition that involves the following sequence. Reduction of PI3K activity initially leads to reduced AKT phosphorylation and activity which in turn, increases transcriptional activation of RTK genes IR, IGFR1, HER2, HER3, EphA7 and EphA10. Increased RTK protein expression, particularly, IR and IGFR1, likely drives the kinetics in favor of their homo- or hetero-dimerization and activation which in turn, leads to activation of MEK-ERK and PI3K-AKT pathways. Increased activation of these pathways lead to decreased apoptosis leading to increased cell viability and resistance. A critical step in this model that predicts resistance to PI3K inhibition is the compensatory activation of a parallel pathway driven by MEK-ERK (Fig 6C). The result that the combined inhibition of PI3K and MEK is sufficient to abrogate the MEK-ERK rebound (Fig 6D) seen in response to PI3K single agent inhibition supports this hypothesis. Interestingly, while PI3K inhibition leads to compensatory increase in MEK-ERK activation, MEK single agent inhibition does not lead to a corresponding increase in PI3K-AKT activation suggesting independent regulation of the MEK-ERK pathway in MCA cells. The reasons for the lack of compensatory increase in PI3K-AKT signaling in response to MEK inhibition is unclear. Nonetheless, our studies strongly suggest that targeting PI3K pathway effectors in MCA is unlikely to be successful unless combined with MEK-ERK pathway inhibition.

The cellular effectors of the PI3K pathway that help induce expression of RTKs (particularly, IR and IGFR1) in response to PI3K inhibition are not definitively known. Based on published studies, one plausible mechanism could be through the actions of FOXO proteins that are direct targets of AKT. In breast cancer lines, resistance to AKT inhibition is associated with increased expression of RTKs HER3, IR and IGF1R [40]. In these lines, inhibition of AKT phosphorylation relieves inhibition of FOXO proteins thereby facilitating their nuclear retention. Increased FOXO activation leads to transcriptional upregulation of RTKs [40]. Whether FOXO proteins help mediate resistance to PI3K inhibition in MCA cells is currently being investigated.

Currently, there are many different MEK inhibitors that are in various stages of clinical development [19]. In addition, clinical effects of combined therapies targeting the PI3K/AKT and RAS/RAF/MEK/ERK pathway are also being evaluated [41]. Results of phase I MEK and PI3K combination therapy suggest that the dosing and treatment regimen would have to be optimized to achieve a balance between efficacy and toxicity. Nonetheless, these agents appear to have a reasonable toxicity profile when used in combination [42]. The nuances in MEK-ERK and PI3K-AKT pathways in KRAS mutant MCAs revealed by our studies underscore the fact that these signaling pathways and the restraints placed on these pathways by negative feedback circuits need to be understood before translation into treatment options. In addition, the effectiveness of MEK and PI3K inhibitors in reducing peritoneal tumor growth will need to be tested in MCA patient-derived xenograft mouse models as such models more faithfully recapitulate the tumor heterogeneity seen in human cancers than cell line-derived xenograft models.

In conclusion, our results show, for the first time, that concomitant inhibition of MEK and PI3K-driven pathways downstream of mutant KRAS leads to substantial reduction of MUC2 and shrinkage of MCA tumors in vitro and in vivo. As PI3K single agent inhibition also relieves negative feedback loops and induction of MEK-driven compensatory pathways, dual attenuation of PI3K and MEK-driven pathways will be needed to achieve measurable and durable response. As there are no targeted treatment interventions for peritoneal mucinous malignancies such as MCA or PMP, these studies have a significant translational potential.

## Supporting information

S1 Fig MCA cells are sensitive to PI3K inhibition at 48 hours. LS174T and RW7213 cells were treated with increasing concentrations of the PI3K inhibitor, Pictilisib, for 48 hours. Each data point has n = 6. Error bars indicate standard error of the mean. Both cell lines, though sensitive to PI3K inhibition at 48 hours, were resistant by 72 hours (i.e. the data (10 data points for each assay, n = 6 for each point) could not be fit to a 4 parameter logistic/sigmoidal dose response curve with an r^2^>0.9 and a negative Hill slope) (data not shown).
(TIF)

S2 Fig Reduction in viability of MCA cells treated with PI3K inhibitor and lapatinib (inhibitor of EGFR and ErbB2). RW7213 cells was treated with Lapaitinib (blue) and PI3Ki (red) as single agents or in combination (green) in a fixed ratio for 72 hours. Each data point is the average of an n = 6. Error bars indicate standard error of the mean.
(TIF)

S3 Fig Knockdown of wild type KRAS in MCA cells in vitro. Knockdown of wildtype KRAS in LS174T cells was assessed by western blots that were probed with an anti-KRAS antibody that can bind to both wildtype and mutant KRAS (top left panel) or an anti-KRASG12D antibody that binds to mutant G12D but not wildtype KRAS protein (top right panel). The blot on the top left shows a reduction in total KRAS protein levels (red arrow) and the blot on the top right panel shows that mutant KRAS G12D levels remain unaltered (green arrow). Expression levels were quantified by densitometry and percent protein expression in DOX-treated samples relative to untreated controls (normalized to actin) are shown below the blots. Abbreviations: DOX, doxycycline; d, days.
(TIF)

S4 Fig Induction of apoptosis in response to combined inhibition of MEK and PI3K in MCA in vitro. Western blots of proteins isolated from LS174T and RW7213 cells treated with either Cobimetinib, Pictilisib or both were probed with anti-caspase 3 antibody that detects cleaved (but not full length) caspase 3, a hall mark of apoptosis. Cleaved caspase 3 (Cl. csp3) levels were increased in MCA cell lines, LS174T and RW7213, in combined treatment with Cobimetinb and Pictilisib than with single agent treatment. Fold change in protein levels are relative to vehicle-treated controls (all normalized to β-actin).
(TIF)

S1 Table Short hairpin RNA (shRNA) sequences. (DOCX)
