# Supplementary figures and images for: Bilateral blockade of MEK- and PI3K-mediated pathways downstream of mutant KRAS as a treatment approach for peritoneal mucinous malignancies
Source: PLoS One. 2017 Jun 22;12(6):e0179510. doi: 10.1371/journal.pone.0179510 (PMC5480880; doi:10.1371/journal.pone.0179510)

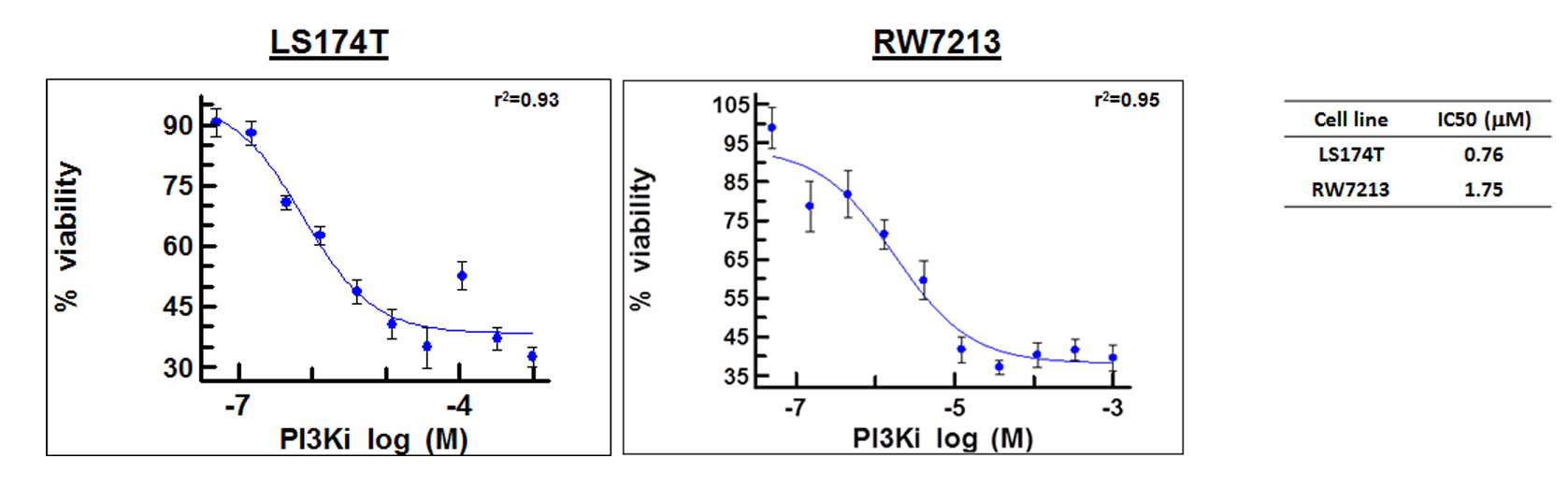

Supplement: S1 Fig — LS174T and RW7213 cells were treated with increasing concentrations of the PI3K inhibitor, Pictilisib, for 48 hours. Each data point has n = 6. Error bars indicate standard error of the mean. Both cell lines, though sensitive to PI3K inhibition at 48 hours, were resistant by 72 hours (i.e. the data (10 data points for each assay, n = 6 for each point) could not be fit to a 4 parameter logistic/sigmoidal dose response curve with an r2>0.9 and a negative Hill slope) (data not shown). (TIF) [file pone.0179510.s001.tif]

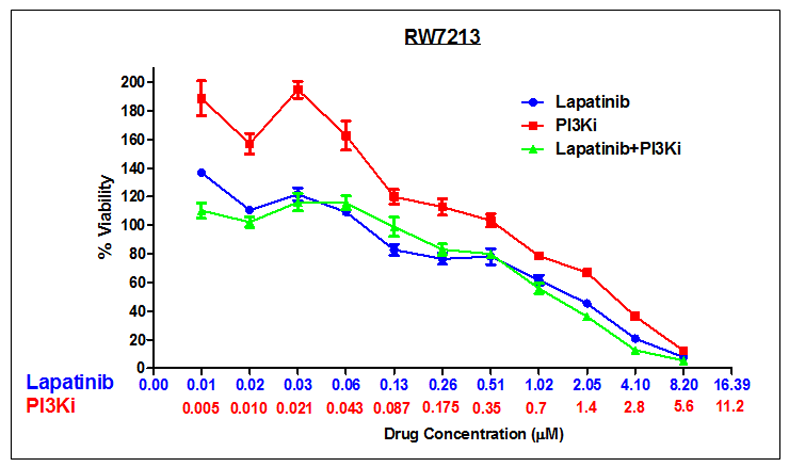

Supplement: S2 Fig — RW7213 cells was treated with Lapaitinib (blue) and PI3Ki (red) as single agents or in combination (green) in a fixed ratio for 72 hours. Each data point is the average of an n = 6. Error bars indicate standard error of the mean. (TIF) [file pone.0179510.s002.tif]

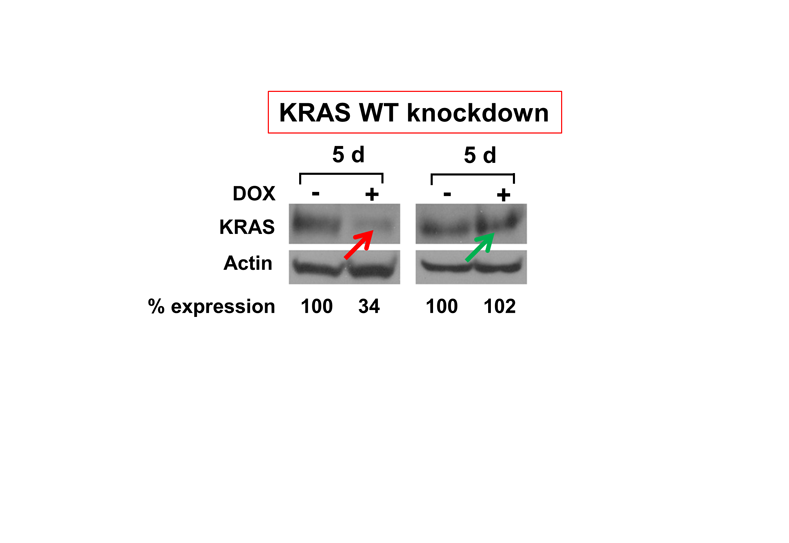

Supplement: S3 Fig — Knockdown of wildtype KRAS in LS174T cells was assessed by western blots that were probed with an anti-KRAS antibody that can bind to both wildtype and mutant KRAS (top left panel) or an anti-KRASG12D antibody that binds to mutant G12D but not wildtype KRAS protein (top right panel). The blot on the top left shows a reduction in total KRAS protein levels (red arrow) and the blot on the top right panel shows that mutant KRAS G12D levels remain unaltered (green arrow). Expression levels were quantified by densitometry and percent protein expression in DOX-treated samples relative to untreated controls (normalized to actin) are shown below the blots. Abbreviations: DOX, doxycycline; d, days. (TIF) [file pone.0179510.s003.tif]

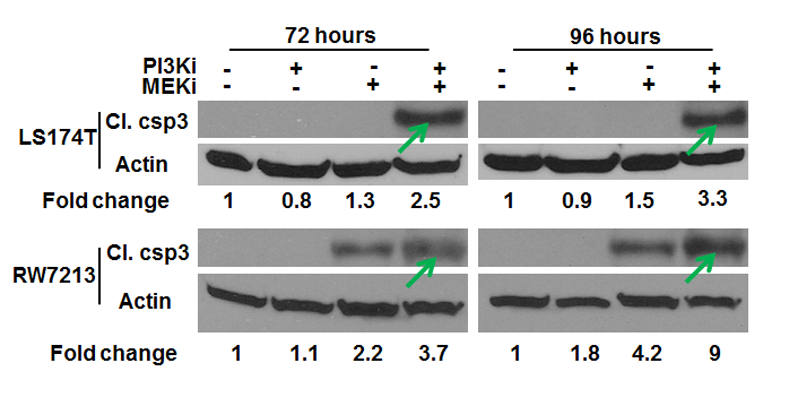

Supplement: S4 Fig — Western blots of proteins isolated from LS174T and RW7213 cells treated with either Cobimetinib, Pictilisib or both were probed with anti-caspase 3 antibody that detects cleaved (but not full length) caspase 3, a hall mark of apoptosis. Cleaved caspase 3 (Cl. csp3) levels were increased in MCA cell lines, LS174T and RW7213, in combined treatment with Cobimetinb and Pictilisib than with single agent treatment. Fold change in protein levels are relative to vehicle-treated controls (all normalized to β-actin). (TIF) [file pone.0179510.s004.tif]
