# Supplementary material for: Bilateral blockade of MEK- and PI3K-mediated pathways downstream of mutant KRAS as a treatment approach for peritoneal mucinous malignancies
Source: PLoS One. 2017 Jun 22;12(6):e0179510. doi: 10.1371/journal.pone.0179510 (PMC5480880; doi:10.1371/journal.pone.0179510)
Supplement: S1 Table — (DOCX) [file pone.0179510.s005.docx]

**S1 Table. Short hairpin RNA (shRNA) sequences**

|  | **Gene Name** | **shRNA sequence** | **Genbank Accession numbers (targeted Sequences)** |
| --- | --- | --- | --- |
| 1 | KRAS  WT* and mutant | TGCTGTTGACAGTGAGCGCCCTATGGTCCTAGTAGGAAATTAGTGAAGCCACAGATGTAATTTCCTACTAGGACCATAGGTTGCCTACTGCCTCGGA | NM_033360 (510...531)  [NG_001154](http://www.ncbi.nlm.nih.gov/nuccore?term=NG_001154) (547...568)  [NM_004985](http://www.ncbi.nlm.nih.gov/nuccore?term=NM_004985) (510...531) |
| 2 | KRAS mutant (G12D) | TGCTGTTGACAGTGAGCGCAGTTGGAGCTGATGGCGTAGGTAGTGAAGCCACAGATGTACCTACGCCATCAGCTCCAACTATGCCTACTGCCTCGGA | NM_033360(216-236)**  NM_004985(216-236)** |
| 3 | KRAS  WT | TGCTGTTGACAGTGAGCGAGGCTATATTTACATGCTACTATAGTGAAGCCACAGATGTATAGTAGCATGTAAATATAGCCCTGCCTACTGCCTCGGA | NM_033360 (1644-1664)  NM_004985(1520-1540) |
| 4 | HRAS | TGCTGTTGACAGTGAGCGCCCAGTACAGGGAGCAGATCAATAGTGAAGCCACAGATGTATTGATCTGCTCCCTGTACTGGTTGCCTACTGCCTCGGA | [NM_005343](http://www.ncbi.nlm.nih.gov/nuccore?term=NM_005343) (471-.492)  NM_176795(470-489)  NM_001130442(470-489) |
| 5 | NRAS | TGCTGTTGACAGTGAGCGCGCAAGTCATTTGCGGATATTATAGTGAAGCCACAGATGTATAATATCCGCAAATGACTTGCTTGCCTACTGCCTCGGA | [NM_002524](http://www.ncbi.nlm.nih.gov/nuccore?term=NM_002524) (514-535) |

Sequences highlighted in green were designed to bind to nucleotide sequences (shown in the fourth column) within the target and sequences in red font indicate the hairpin loop within the miR30 backbone of the pINDUCER vector (see Fig. 1A)

*Abbreviation: WT, wildtype

**The KRAS mutant shRNA targets sequences 216 to 236 within exon 2 of the mutant KRAS gene (NM-033360 and NM-004985 are wildtype KRAS sequences which are identical to mutant KRAS except at codon 12 where the Glycine in the wildtype is altered to Aspartate in the mutant)
